# Promotion of Early Gut Colonization by Probiotic Intervention on Microbiota Diversity in Pregnant Sows

**DOI:** 10.3389/fmicb.2017.02028

**Published:** 2017-10-20

**Authors:** Katarina Veljović, Miroslav Dinić, Jovanka Lukić, Sanja Mihajlović, Maja Tolinački, Milica Živković, Jelena Begović, Igor Mrvaljević, Nataša Golić, Amarela Terzić-Vidojević

**Affiliations:** ^1^Laboratory for Molecular Microbiology, Institute of Molecular Genetics and Genetic Engineering, University of Belgrade, Belgrade, Serbia; ^2^Centre for Development and Production, Veterinary Station “Koker”, Adaševci, Serbia

**Keywords:** probiotic, pathogen exclusion, neonatal piglets, microbiota diversity, DGGE

## Abstract

The aim of this work was to design a novel mixed probiotic culture for piglets and to evaluate its beneficial effect on the piglets’ gut health. The possible mechanisms of probiotic activity, such as adhesion, competitive pathogen exclusion and influence on gut microbiota diversity were determined. Mixed probiotic starter culture is composed of three thermophilic lactic acid bacteria (LAB) strains: *Lactobacillus helveticus* BGRA43, *Lactobacillus fermentum* BGHI14 and *Streptococcus thermophilus* BGVLJ1-44. The strains BGVLJ1-44 and BGRA43 showed good technological properties (fast milk curdling, strong proteolytic activity). In addition, the strain BGVLJ1-44 produces exopolysaccharide (EPS), BGHI14 is heterofermentative LAB strain with significant immunomodulatory effect, while the strain BGRA43 showed strong antimicrobial activity against different pathogens and exhibited significantly higher level of adhesion to Caco-2 cells comparing to other two strains. Both lactobacilli strains BGRA43 and BGHI14 (*p* < 0.05), as well as probiotic combination (*p* < 0.01) significantly reduced the adhesion of *Escherichia coli* ATCC25922 to Caco-2 cells, while the strains BGVLJ1-44 (*p* < 0.01) and BGRA43 (*p* < 0.05) significantly reduced adhesion of *Salmonella* 654/7E (veterinary isolate). The results of farm trial revealed that treatment of sows with new fermented dairy probiotic influenced the piglets’ gut colonization with beneficial bacteria and reduced the number of enterobacteriaceae in litters from some treated sows (no significant due to high variability among animals). Finally, this is the first study reporting that the treatment of sows with probiotic combination resulted in the improved microbiota diversity in neonatal piglets.

## Introduction

The establishment of gut microbiota immediately after birth is a prerequisite for the healthy animals. In the first days after birth animals are prone to bacterial infections from the environment and it is extremely important for development of their immune system. Probiotics, defined as “live microorganisms, which when administered in adequate amounts confer a health benefit on the host” (FAO-WHO, 2006), could be used as mono- or mixed cultures. The probiotic bacteria have a number of beneficial effects, including immunomodulation, pathogen exclusion and positive influence on maintenance of gut microbiota composition. Probiotics can decrease the incidence and severity of diarrhea caused by ETEC (Kyriakis et al., 1999). Lately, the numbers of studies are intensively engaged in the development of probiotic products for animals, based on the use of lactic acid bacteria (LAB), *Bacillus* sp. or yeasts together with their associated products such as organic acids and exopolysaccharides. The *in vitro* adhesion of ETEC to porcine erythrocytes was reduced by exopolysaccharides (EPS) produced by *Lactobacillus reuteri* (Wang et al., 2010). Similarly, the protective role of the EPS-producing *Lactobacillus paraplantarum* BGCG11 on HT29-MTX challenged with *Escherichia coli* was shown previously, as well as the ability of EPS-SJ producing strain *Lactobacillus paracasei* subsp. *paracasei* BGSJ2-8 to reduce the *E. coli* ATCC25922’s association to Caco-2 cells (Živković et al., 2015, 2016). In addition, it has been shown that feed fermentation can deliver such a combination of probiotic lactobacilli that prevent pathogen adhesion (van Winsen et al., 2001; Yang et al., 2015). Due to the synergistic effects, the mixed-probiotic cultures can be more active against different pathogens, as well as in term of improving the colonic health and nutrition (Umesaki and Setoyama, 2000; Timmerman et al., 2004; Collado et al., 2007).

The increased interest in use of probiotics in livestock has arisen since 2006 when the use of antibiotics as growth promoting factors in livestock has been banned by European Union (Castanon, 2007). The most desirable probiotics features in livestock production are generally related to growth promotion in farm animals, improvement of nutrient digestibility, protection from intestinal infections, immunomodulation, and replacement of beneficial microorganisms that are absent from intestinal tract due to disbiosis (Cho et al., 2011). It has been increasingly acknowledged that ingestion of beneficial microorganisms immediately after birth has a remarkable impact on the piglets’ postnatal development, referred to as ‘microbial imprinting’ (Zoetendal et al., 2001; Mazmanian et al., 2005; Konstantinov et al., 2006). On the other hand, in that period piglets might be infected by pathogenic bacteria causing intestinal infections accompanied by diarrhea, weight loss and even mortality. In order to assess the efficacy of probiotics and to ensure the permanent or transient colonization of piglets’ intestinal mucosa, the most appropriate time of probiotics’ applications should be considered. Ideally, bacterial ‘imprinting’ should happen in very early life, meaning that the piglets should be exposed to a beneficial microbiota at the time of birth in order to be protected against environmentally acquired pathogens (Kenny et al., 2011). On the other hand, for optimal use in a farm setting, probiotics need to be cost-effective. Thus, the most suitable and cost effective way of probiotic application on pig farms might be the treatment of sows, providing the neonatal piglets with desirable microbes immediately after birth, together with the passive immunity from the treated mothers. Relatively few studies reported the application of LAB in sows. It was observed that administration of *L. johnsonii* XS4 toward the end of pregnancy and during lactation had positive effects on the sows and piglets, including a significant increase of IgG levels in serum (Wang and Donovan, 2015; Yang et al., 2015).

In this study probiotic and technological properties of two human intestinal isolates *Lactobacillus helveticus* BGRA43 and *Lactobacillus fermentum* BGHI14 and one dairy natural isolate from artisanal yogurt *Streptococcus thermophilus* BGVLJ1-44 were evaluated. The strain BGRA43 exhibits wide range of antimicrobial activity against number of pathogens (Strahinic et al., 2013), BGHI14 was shown to have immunomodulatory potential in a way to burst immune response (Lukic et al., 2013), while BGVLJ1-44 produces ropy exopolysaccharide (EPS) and has good technological characteristics as dairy starter culture. Here we present the potential of the strains for adhesion to epithelial intestinal cells and pathogen exclusion (*in vitro*), as well as the ability of the mixed probiotic culture to influence the gut microbiota of weaning piglets and to prevent the occurring of intestinal infections (*in vivo* in farm trial). According to our knowledge, this is the first study reporting that the probiotic intervention on sows resulted in the improved microbiota diversity in neonatal piglets.

## Materials and Methods

### The Strains and Growth Conditions

Three thermophilic lactic acid bacteria species from laboratory collection of Laboratory of Molecular Microbiology, Institute of Molecular Genetics and Genetic Engineering, University of Belgrade, Serbia were used in this study: *Lactobacillus helveticus* BGRA43 (Acc. No. LN909514) isolated from human intestinal tract (Strahinic et al., 2013), *Lactobacillus fermentum* BGHI14 (Acc. No. LN909513) isolated from newborn feces of a breast-fed infant (Lukic et al., 2013) and *Streptococcus thermophilus* BGVLJ1-44 (Acc. No. LN909512) isolated from an artisanal yogurt produced from uncooked cow’s milk.

For the cultivation of *S. thermophilus* BGVLJ1-44 liquid and solid M17 medium (Merck GmbH, Darmstadt, Germany), supplemented with glucose (0.5% w/v) (GM17) was used, while for the cultivation of strains *L. fermentum* BGHI14 and *L. helveticus* BGRA43 liquid and solid MRS medium (Merck, GmbH) was used. The strains were grown at 37°C, in anaerobic condition using Anaerocult A (Merck). Solid medium plates were prepared by adding agar (1.7% w/v, Torlak, Belgrade) into each medium broth. *Esherichia coli* ATCC25922 and *Salmonella typhimurium* Enteriditis 654/7E (veterinary isolate, the Collection of Scientific Institute of Veterinary Medicine, Novi Sad, Serbia) were cultivated in Luria-Bertani broth (LB) at 37°C.

### Physiological, Biochemical and Technological Characterization of Strains

The strains *L. helveticus* BGRA43, *L. fermentum* BGHI14 and *S. thermophilus* BGVLJ1-44 were subjected to a set of tests as follows: growth at different temperatures (15°C, 30°C and 45°C); growth in broth with 2% NaCl (w/v); L-arginine and esculine hydrolysis, the use of citrate as energy source (Kempler and Mckay, 1981); CO_2_ production from glucose in reconstituted MRS broth tubes containing inverted Durham bells; the production of acetoin by the Voges-Proskauer test (Zourari et al., 1991); diacetyl production was tested only qualitatively. After overnight incubation of 11% reconstituted skimmed milk (RSM) at 37°C, 1 ml of coagulated milk was mixed with 0.1 g of creatinine (Alfa Aesar, GmbH & Co KG, Karlsuhe, Germany) and 1 ml of 30% NaOH (w/v). Diacetyl production was indicated by the formation of a red halo at the top of the tubes after 2 h of incubation at room temperature. EPS production was detected visually on MRS or GM17 agar plates, depending on the strain, as long strands when the colonies were extended with an inoculation loop (Nikolic et al., 2012). Speed curdling was determined visually by time measuring from the moment of inoculation of 11% sterile RSM with each single starter culture to the moment of curd forming at the incubation temperature of 42°C. Test in litmus milk was prepared in RSM with litmus indicator. After overnight incubation of inoculated RSM at 37°C the changes of color and structure of litmus milk were detected according to Cappuccino and Sherman (2010) as follows: A-acid production (red ring); C-curdling (milk curdled due to lactose fermentation) and R-reduction of H^+^ ions (white color). Test of surviving of the strains BGRA43, BGHI14 and BGVLJ1-44 in MRS and GM17 broth after heating at 63.5°C for 30 min was performed too. Aggregation ability of tested starter cultures was detected visually after shaking of tubes with inoculated MRS or GM17 broth that were previously incubated overnight at 37°C.

The proteolytic activity of the isolates BGRA43, BGHI14 and BGVLJ1-44 were assayed as previously described (Kojic et al., 1991). The collected fresh cells (10 mg with an approximate density of 10^10^ cells/ml) were resuspended in 0.1 M of sodium phosphate buffer with pH 6.8 and mixed in a 1:1 ratio with 5 mg/ml of α_s1_-, β- and κ- casein respectively (Sigma, St. Louis, MO, United States) dissolved in the identical buffer. The mixtures were incubated for 3 h at 37°C. The degradation of α_s1_-, β- and κ- casein was analyzed on 12.5% sodium dodecyl sulfate-polyacrylamide gel electrophoresis (SDS-PAGE). The level of α_s1_-, β- and κ- casein degradation was quantified by ImageQuant software (Molecular Dynamics GmbH, Krefeld, Germany).

### Survival of the Strains in Simulated Gastrointestinal Tract Transit

Survival of probiotic strains after the chemically simulated gastrointestinal tract (GIT) transit was essentially performed as described previously (Nikolic et al., 2012), with minor modifications. The strains were grown in appropriate medium for 24 h and cultures were harvested by centrifugation (10,000 × *g* for 10 min), washed twice with 0.85% NaCl and concentrated 10-times in reconstituted (10%) sterilized skimmed-milk (Difco, Becton Dickinson, Franklin Lakes, NJ, United States) or in saline buffer (0.85% NaCl). Afterward, bacterial suspensions were diluted 10-times with gastric juice (GJ: 125 mM NaCl, 7 mM KCl, 45 mM NaHCO_3_, 0.3% Pepsine [Sigma, St. Louis, MO, United States] adjusted to pH 2 with HCl), incubated for 90 min at 37°C in aerobic conditions under shaking (≈180 rpm). Then, bacterial suspensions were centrifuged (2050 × *g*, 15 min), resuspended in duodenal juice (DJ: 1% bovine bile [Sigma] adjusted with 10 M NaOH to pH 8.0) and incubated for 10 min at 37°C in anaerobic condition using Anaerocult A (Merck). Finally, harvested cell suspensions were resuspended in intestinal juice (IJ: 0.3% bovine bile, 0.1% Pancreas acetone powder porcine Type I [Sigma], pH 8.0) and incubated for 120 min at 37°C in anaerobic conditions. Two samples were collected during IJ challenge, after 60 and 120 min. Determination of viable counts was carried out in the initial cultures, as well as after each of the challenges. Serial dilutions of the samples were made in 0.85% NaCl and 10 μl were spotted onto an appropriate medium. Plates were incubated for 24 h at 37°C and results were expressed as CFU/ml. The percentage of survival was calculated from the viable counts recovered after each chemically simulated GIT step with respect to the initial counts (% CFU recovered bacteria/CFU initial bacteria). The experiments were performed in triplicate.

### Adhesion of the Strains to Caco-2 Intestinal Epithelial Cells

Adhesion to Caco-2 intestinal epithelial cells (IEC) was performed as previously described (Nikolic et al., 2012). The colonocyte-like cell line Caco-2 was used to determine the adhesion ability of bacterial strains. Caco-2 was purchased from the European Collection of Cell Cultures (ECACC No. 86010202). The culture and maintenance of the cells was carried out following standard procedures (Sánchez et al., 2010) using DMEM medium for Caco-2. Media and reagents were purchased from Sigma (St. Louis, MO, United States). Caco-2 cells (passage 5–6) were seeded in 24-well plates and cultivated until a confluent differentiated state was reached. For adhesion experiments, 13 ± 1 day-old cellular monolayers were used. All strains were cultured for 24 h and after washing twice with PBS solution were resuspended in the corresponding cell-line media without antibiotics at a concentration of about 10^8^ CFU/ml. Cellular monolayers were also carefully washed and bacterial suspensions were added at a ratio of about 10:1 (bacteria : eukaryotic cell). Adhesion experiments were carried out for 1 h at 37°C, 5% CO_2_ and, afterward, wells were gently washed to release unattached bacteria before proceeding with the lysis of cellular monolayers using 0.25% Trypsin–EDTA solution (Sigma, St. Louis, MO, United States). Dilutions of samples, before and after adhesion, were made in Ringer’s solution and bacterial counts were performed on GM17 and MRS plates. The adhesion was calculated as: % CFU adhered bacteria/CFU added bacteria. Experiments were carried out in two replicated plates and in each plate three wells were used per sample.

### Reduction of *E. coli* ATCC25922 and *Salmonella* 654/7E Adhesion to Caco-2 Cells in Presence of the Strains

The reduction of adhesion of *E. coli* ATCC25922 and *Salmonella* 654/7E strains in the presence of probiotic strains was tested on Caco-2 cells as described previously (Živković et al., 2016). The capability of *E. coli* ATCC25922 and *Salmonella* 654/7E strains to be associated to the Caco-2 cells in the presence and absence of probiotic strains, as well as in the presence of probiotic combination were tested. Bacterial cultures were washed twice with PBS and resuspended in DMEM without antibiotics at a concentration of ∼1 × 10^7^ CFU/ml; this number was corroborated by plate counting in the agar medium specific for each bacterium. The bacterial suspensions containing one of the pathogens or a combination of pathogens and probiotic strains (ratio 1:1) were independently added to the Caco-2 monolayers at a ratio of 10:1, (bacteria : eukaryotic cells; in the case of pathogens – probiotic combination, each bacterial type was added at a ratio of 10:1) and incubated at 37°C, with 5% CO_2_ for 1 h. Afterward, the monolayers were gently washed twice with PBS to remove the unattached bacteria, and the eukaryotic cells were released using 0.25% Trypsin–EDTA solution (Sigma, St. Louis, MO, United States). The samples were diluted in 0.85% (w/v) NaCl buffer and plated to LB medium to enumerate the associated *E. coli* ATCC25922 and *Salmonella* 654/7E strains. The percentage of *E. coli* ATCC25922 and *Salmonella* 654/7E strains association was calculated as follows: 100 × CFU/ml bacteria associated/CFU/ml bacteria added (the dilution of bacteria was taken into account). Each combination was tested in triplicate. To determine the capability of probiotic strains to decrease the association of *E. coli* ATCC25922 and *Salmonella* 654/7E strains to Caco-2 monolayers, data were referred to that obtained with the *E. coli* ATCC25922 and *Salmonella* 654/7E strains alone, respectively (i.e., 100% association). Each combination of pathogens and probiotic strains, and the pathogens strain alone, respectively, were tested in three replicates.

### Design of Probiotic Culture

Preparation of fermented probiotic culture includes the milk fermentation process by use of starter and probiotic bacteria in defined ratio. Incubation of inoculated milk was carried at 42°C for approximately 5 h until the pH 4.8 was reached. After that, fermented milk was transferred to the refrigerator at 4°C where the pH of fermented milk is further slowly lowered to 4.6 during cooling. The pH value and total viable count of probiotic bacteria was estimated immediately after the inoculation of milk as well as each hour during first 5 h of the milk fermentation and after 24 h, 7, 14, and 21 days of storage of fermented dairy probiotic at 4°C. Experiments are performed in three independent measurements.

### Farm Trial Study Design

The effect of the probiotic combination (including strains: BGVLJ1-44, BGHI14 and BGRA43) on the health status of farm animals was monitored *in vivo* in farm trial. The experiments were performed in accordance to the European convention for protection of vertebrate animals used for experimental and other scientific purposes (Directive 2010/63/EU) as well as International Guiding Principles for Biomedical Research involving Animals (C.I.O.M.S., c/o WHO, CH 1211 Geneva 27, Switzerland), and approved by Ethical Committee of Faculty of Biology, University of Belgrade, Serbia; No EK-BF-2013/09). The probiotic strains were prepared in reconstituted (10%) sterilized skimmed-milk (Difco, Becton Dickinson, Franklin Lakes, NJ, United States). Out of 50 pregnant sows 25 were treated with probiotic combination for 10 days before predicted farrowing term (at a time when the natural immunity of pregnant sows usually declines). Pregnant sows have been received the 200 ml of mixed probiotic culture (10^8^ CFU/ml) every day during the first morning feeding. In parallel, 25 control sows, not treated with the probiotic, were followed. Before and after the probiotic treatment, the general health of treated and control sows and the total number of bacteria (LAB and *Enterobacteriaceae*) in piglets’ faces was followed as described previously (Anonymous, 1996; Terzic-Vidojevic et al., 2007).

### Denaturing Gradient Gel Electrophoresis (DGGE) Analysis

Extraction of bacterial DNA from fecal samples was done using the ZR Genomic DNA Tissue Mini Prep kit (Zymo Research, Irvine, CA, United States). PCR reaction with isolated bacterial DNA as a template, denaturing gradient gel electrophoresis (DGGE) analysis and gel manipulation after electrophoresis was performed essentially as described previously (Lukic et al., 2013). The dominant bacterial communities were determined by PCR using primers U968-GC and L1401-r (Invitrogen, Paisley, United Kingdom) complementary to the V6 to V8 region of eubacterial 16S rDNA (Randazzo et al., 2006), while lactobacilli diversity was detected by lactobacilli-specific primers Lab-0159f and Uni-0515-GCr (Invitrogen, Paisley, United Kingdom) (Lukic et al., 2013). The reaction was performed in thermal cycler Gene AmpR PCR System 2700 (Applied Biosystems, Foster City, CA, United States). Bacterial DNA was then set on denaturing gradient gel prepared according to Lukic et al. (2013) and glass plates for DGGE apparatus DGGE-2001 (C.B.S. Scientific, San Diego, CA, United States) were used.

### Enterotoxigenic *Escherichia coli* and *Salmonella* sp. Detection

To detect possible presence of the most common intestinal pathogens in piglets’, PCR reactions were performed using as a template 100 ng of DNA extracted from fecal samples [as described in Section “Denaturing Gradient Gel Electrophoresis (DGGE) Analysis”]. Primers used in the study were complementary to *eltA* gene and *invA* gene, specific for enterotoxigenic *Escherichia coli* (Youmans et al., 2014) and *Salmonella* sp. (Li and Chen, 2013), respectively. PCR reactions were prepared using 10 pmols of each primer, 0.2 mM dNTP and 0.6 U of *Taq* Polymerase in a volume of 30 μl and run under the following conditions: 94°C for 5 min and then 35 cycles of 94°C for 30 s, 57°C for 60 s and 72°C for 30 s with a final extension at 72°C, 7 min.

### Statistical Analysis

Differences between treatments were examined for significance by Student’s *t*-test after analysis of variance. For comparison of proportions of individual bands on DGGE profiles between piglets from different treatment groups, Pearson’s chi-squared test (χ2) was applied. Wilcoxon Signed Ranks test was used to compare the number of total bands in DGGE profiles in piglets with the number of bands in their mother sows. *P* > 0.05 was considered to be statistically insignificant.

## Results

### *In Vitro* GIT Survival of Probiotic Strains

**FIGURE 1:**
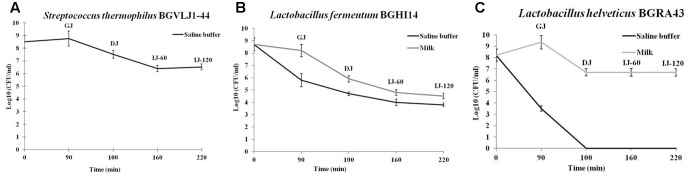
Survival of the strains *Streptococcus thermophilus* BGVLJ1-44 **(A)**, *Lactobacillus fermentum* BGHI14 **(B)** and *Lactobacillus helveticus* BGRA43 **(C)** in simulated conditions of the gastrointestinal tract. GJ – time point when samples were taken from chemically simulated gastric juice; DJ – time point when samples were taken from chemically simulated duodenal juice; IJ-60 and IJ-120 – the time points (60 and 120 min, respectively) when samples were taken from chemically simulated intestinal juice. Values represent the means of three experiments. Standard deviations are shown.

The survival of probiotic strains *S. thermophilus* BGVLJ1-44, *L. fermentum* BGHI14 and *L. helveticus* BGRA43 after chemically simulated GIT transit was tested. The results revealed that only the strain BGVLJ1-44 successfully survived the passage through simulated GIT conditions in saline buffer, while the strains *L. helveticus* BGRA43 survived in simulated GIT conditions only when administered in milk (**Figure 1**). The results showed that the number of viable cells administered in milk after 90 min was similar to that at the beginning of the experiment in all probiotic strains, indicating that all strains were resistant to acidic conditions. However, 10 min exposure to high bile salts concentration decreased the survival of the strain BGHI14 both in saline (Δlog CFU/ml 1.1) and milk (Δlog CFU/ml 2.3), while the strain BGRA43 survived only when administred in milk (Δlog CFU/ml 2.65). Finally, 120 min exposure to lower bile salt concentration (0.3%) and pancreatic digestion considerably reduced the viability of BGHI14 (Δlog CFU/ml from 0.9 to 1.1 in saline buffer and milk, respectively), as well as the number of viable cells of BGVLJ1-44 (Δlog CFU/ml 1). Interestingly, the number of viable cells of BGRA43 in milk remained the same. The best survival rate showed BGRA43 (3.2%; Δlog CFU/ml 1.5) in milk, while the survival rate of BGVLJ1-44 was 1%; Δlog CFU/ml 2 in saline. The lowest survival rate showed BGHI14 (0.01%; Δlog CFU/ml 4.2 when administered in milk and 0.001%; Δlog CFU/ml 4.9 when administered in saline) (**Figure 1**).

### Characterization of Technological Properties of the Strains and Preparation of Fermented Probiotic

Since the results of *in vitro* GIT survival showed that the best survival rate of the strains was obtained in milk, the strains *S. thermophilus* BGVLJ1-44, *L. fermentum* BGHI14 and *L. helveticus* BGRA43 were used as starter cultures for preparation of fermented dairy probiotic. The general physiological, biochemical and technological characteristics of the strains are summarized in **Table 1**. Only *L. fermentum* BGHI14 strain showed ability to grow at all three tested temperatures (15°C, 30°C and 45°C) and none of them grew in broth with 2% NaCl. Hydrolysis of arginine and production of CO_2_ showed *L. fermentum* BGHI14 strain, while production of acetoin and diacetyl were characteristic for *S. thermophilus* BGVLJ1-44 strain. All three LAB strains survived at 63.5°C for 30 min. *L. fermentum* BGHI14 as heterofermentative strain could not coagulate the milk. On the other hand, the strains *S. thermophilus* BGVLJ1-44 and *L. helveticus* BGRA43 showed the ability of fast milk curdling and they completely hydrolyze α_s1-_, β- and κ-casein after 3 h of incubation at 37°C. *S. thermophilus* BGVLJ1-44 strain was an excellent EPS producer (**Table 1**).

**Table 1:** Phenotypic, biochemical and technological characteristics of *Streptococcus thermophilus* BGVLJ1-44, *Lactobacillus fermentum* BGHI14 and *Lactobacillus helveticus* BGRA43 strains.

Feature tested	*Streptococcus thermophilus* BGVLJ1-44	*Lactobacillus fermentum* BGHI14	*Lactobacillus helveticus* BGRA43
Growth at 15°C	–	+	–
Growth at 30°C	+	+	+
Growth at 45°C	–	+	–
Growth in broth with 2% NaCl	–	–	–
Arginine hydrolysis	–	+	–
Esculin hydrolysis	–	±	±
Citrate utilization	–	–	–
Production of CO_2_ from glucose	–	+	–
Production of acetoin	+	–	–
Production of diacetyl	+	–	–
Curdling (h)	6	No curdling	6
Litmus milk	A^1^C^2^R^3^	R	ACR
Survival at 63.5°C for 30 min	±	+	+
Degradation of α_s1-_, β- and κ-casein after 3 h of incubation at 37 °C	α_s1-_ casein (+) β- casein (+) κ-casein(+)	α_s1-_ casein (–) β- casein (–) κ-casein (–)	α_s1-_ casein (+) β- casein (+) κ-casein (+)
Aggregation	–	–	–
Production of exopolysaccharides	+	–	–


**Table 2:** Proportions of bands obtained in DGGE using U968-GC/L1401-r and Lab-0159f / Uni-0515-GCr primer pairs and corresponding p values of Pearson chi-squared (χ2) test.

Band number (U968-GC/L1401-r)	Proportion in piglets from probiotic treated mothers (%)	Proportion in piglets from untreated mothers (%)	*p* (χ^2^)
77	75	0	0.014
76	0	50	0.028
75	75	0	0.014
65	87.5	25	0.03
63	87.5	25	0.03
24	87.5	25	0.03
20	0	50	0.028
11	87.5	0	0.004
10	12.5	100	0.004
Band number (Lab-0159f / Uni-0515-GCr)			
45	12.5	75	0.03
23	0	50	0.028


**FIGURE 2:**
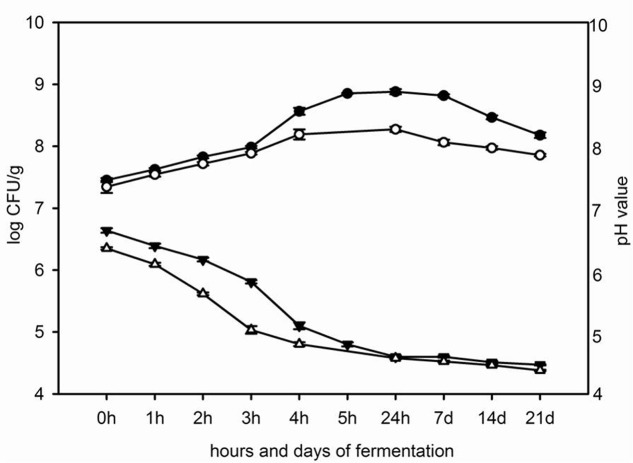
The changes of pH value in milk (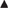) and in whey (△) and the total count of viable mixed probiotic starter cultures in milk (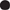) and in whey (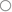) during fermentation for 5 h at 42°C and 21 days of storage at 4°C.

Appart from milk, the rate of acid development was also followed in whey. The pasteurized milk and way were inoculated each with 3% probiotic starter culture. Measuring of pH values during 0, 1, 2, 3, 4, and 5 h of fermentation at 42°C, as well as after 24 h, 7, 14, and 21 days of storage at 4°C was performed. In parallel, total count of viable probiotic LAB was followed (**Figure 2**). The pH of non-inoculated milk and whey were 6.81 and 6.65, respectively.

Probiotic starter culture inoculated in whey decreased pH value faster than probiotic starter culture inoculated in milk. The pH value of fermented whey was 4.80 after 4 h at 42°C and pH of fermented milk was 4.78 after 5 h of incubation at 42°C. During the cooling, the pH values of fermented milk and whey slowly decreased and after 21 days of storage at 4°C the pH values of fermented milk and whey were 4.47 and 4.38, respectively (**Figure 2**).

In parallel, total counts of viable probiotic starter cultures during fermentation of milk and whey were followed. The total number of viable LAB increased in milk and whey from 7.5 log CFU/ml and 7.4 log CFU/ml, respectively, at the beginning of incubation period to 8.9 log CFU/ml in milk and 8.2 log CFU/ml in whey at the end of fermentation at 42°C. After 21 days of storage of the fermented milk and whey the number of probiotic bacteria in fermented milk and whey was 8.2 log CFU/ml and 7.9 log CFU/ml, respectively (**Figure 2**).

### Characterization of the Ability of Probiotic Strains to Reduce the *E. coli* and *Salmonella* Adhesion to Caco-2 Cells

**FIGURE 3:**
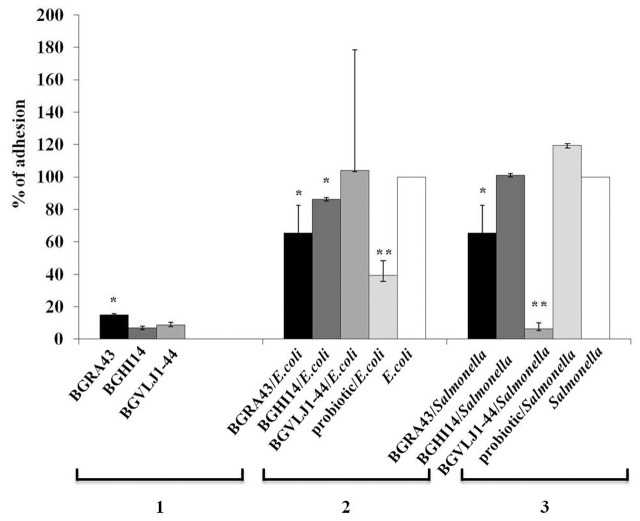
The adhesion of the strains *Streptococcus thermophilus* BGVLJ1-44, *Lactobacillus fermentum* BGHI14 and *Lactobacillus helveticus* BGRA43, as well as *Escherichia coli* ATCC25922 and *Salmonella* 654/7E in the presence and absence of LAB strains and probiotic combination to Caco-2 intestinal epithelial cells. 1: Adhesion of the LAB strains; 2: Adhesion of *E. coli* in the presence and absence of LAB strains and probiotic combination; 3: Adhesion of *Salmonella* 654/7E in the presence and absence of LAB strains and probiotic combination. The values given in the graph represent mean values of three measurements and are expressed in percentages. Statistically significant values are marked with asterisks (^∗^*p* < 0.05, ^∗∗^*p* < 0.01).

In order to determine the ability of the strains *S. thermophilus* BGVLJ1-44, *L. fermentum* BGHI14 and *L. helveticus* BGRA43 to counteract the adhesion of pathogens, the adhesion properties of the probiotic strains to IEC Caco-2 were determined. The strain BGRA43 showed significantly (*p* < 0.05) higher adhesion of 1.7 to 2.1-fold to Caco-2 cells comparing to BGVLJ1-44 and BGHI14, respectively (**Figure 3**).

Further, the potential of probiotic strains BGVLJ1-44, BGHI14 and BGRA43, as well as the mixed probiotic culture, to compete with *E. coli* ATCC25922 and *Salmonella* 654/7E and to reduce their adhesion to Caco-2 cells was studied. The adhesion of *E. coli* ATCC25922 strain was significantly (*p* < 0.05) lower in the presence of BGRA43 (1.5-fold) and BGHI14 (1.2-fold) lactobacilli strains (**Figure 3**). The highest reduction (2.6-fold) of *E. coli* ATCC25922 adhesion to Caco-2 cells was in the presence of the mixed probiotic culture (*p* < 0.01) (**Figure 3**).

In addition, the adhesion of *Salmonella* 654/7E to Caco-2 cells was significantly reduced (16-fold) in the presence of BGVLJ1-44 (*p* < 0.01) and 1.5-fold in the presence of BGRA43 (*p* < 0.05) (**Figure 3**). Interestingly, there was no reduction of *Salmonella* 654/7E adhesion in the presence of BGHI14 and probiotic mixture.

### *In Vivo* Evaluation of the Effect of Probiotic Combination on Neonatal Piglets

Since the results given in Section “*In Vitro* GIT Survival of Probiotic Strains” showed that the number of probiotic bacteria was higher in fermented milk than in whey, the fermented milk containing probiotic strains *L. fermentum* BGHI14, *L. helveticus* BGRA43 and *S. thermophilus* BGVLJ1-44 was used in farm trial.

#### Farm Trial

In order to determine possible protective effect of the mixed probiotic culture on neonatal piglets, the farm trial was performed including 50 pregnant sows (25 sows treated with probiotic combination and 25 control sows). The results showed that none of piglets, from litters of sows treated with the mixed probiotic culture, suffered from diarrhea. In contrast, among piglets from control litters diarrhea occurred sporadically. In addition, body weight of the piglets was followed twice a week during the experimental period and the results were in accordance to the results obtained for the control animals. The experimental animals did not show eating disorders.

**FIGURE 4:**
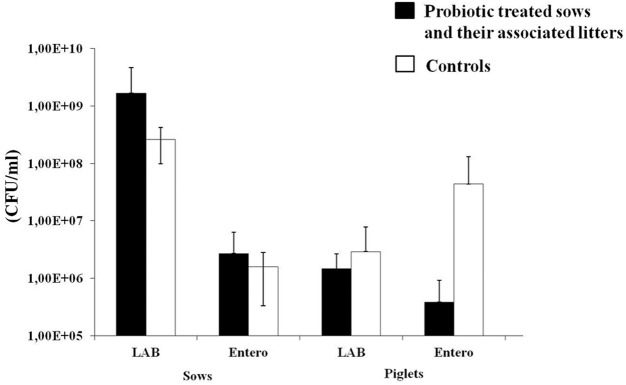
The number (CFU/ml) of lactic acid bacteria (LAB) and *Enterobacteriaceae* in sows (at the beginning of experiment, before probiotic treatment) treated with probiotic combination and control animals, as well as their associated litters (born after the probiotic treatment). Black bars represent sows treated with probiotic combination and their associated litters (as indicated on *x*-axis). White bars represent control sows and their associated litters (as indicated on *x*-axis). Values represent the means of three experiments. Standard deviations are shown.

Importantly, the number of *Enterobacteriaceae* in fecal samples collected from litters of probiotic treated sows was reduced in treated sows compared to the number of *Enterobacteriaceae* in the fecal samples of the litters of untreated sows, although the variability among tested animals was high (**Figure 4**). In order to detect possible presence of ETEC and virulent *Salmonella* sp. strains in the fecal samples from piglets, the PCR analysis with primers specific for the *eltA* gene and *invA* gene, respectively, was performed. However, the bands specific for the *eltA* gene encoding the heat-labile enterotoxin in ETEC and the *invA* gene encoding invasive protein in *Salmonella* sp. were not detected in any of the analyzed samples.

#### DGGE Analysis

**FIGURE 5:**
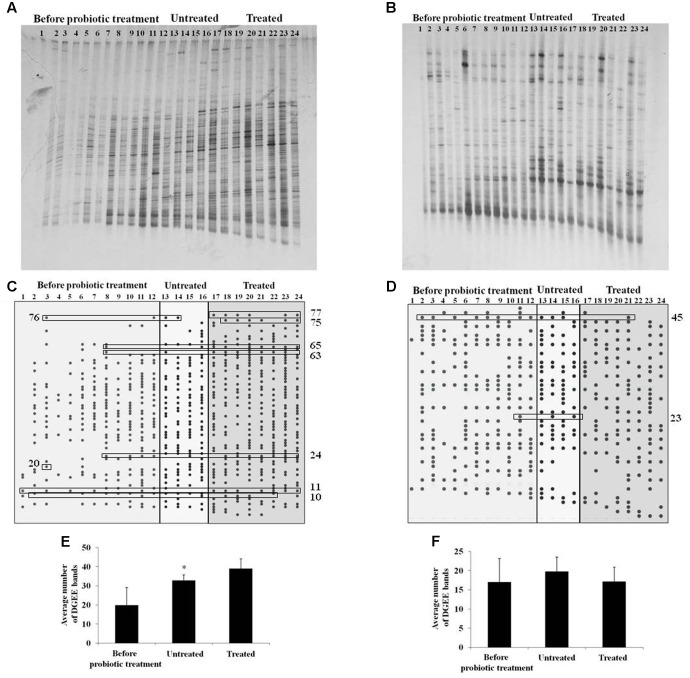
Denaturing gradient gel electrophoresis analysis of microbiota diversity. **(A,B)** DGGE profiles of rDNA amplicons obtained using universal primers **(A)** or *Lactobacillus* sp.-specific primers **(B)** on bacterial DNA isolated from fecal samples of sows (samples 1–12) or litters (13–24) belonging to untreated (13–16) or treated sow (17–24); **(C,D)** Graphical presentation of DGGE bands obtained using universal primers (C) or *Lactobacillus* sp.-specific primers **(D)**. The numbers on the *x*-axis correspond to the numbers of lanes as presented in **(A,B)**. Statistically relevant bands given in **Table 2** are presented in boxes; **(E,F)** The average number of DGGE bands obtained using universal primers **(E)** or *Lactobacillus* sp.-specific primers **(F)** in specific groups (sows before probiotic treatment, litters of untreated sows and litters of treated sows). Statistically significant values compared to the control are marked with asterisks (^∗^*p* < 0.05).

Gut microbiota diversity, as well as LAB diversity in sows before the probiotic treatment and in litters of treated and untreated sows was evaluated by DGGE analysis. Total DNA isolated from fecal samples, as template, and primers complementary to 16S rDNA were used for PCR amplification. In total, 24 randomly chosen DNA amplicons were used (12 from sows before probiotic treatment, four from litters of control untreated sows and eight from litters of sows treated with probiotic combination) (**Figure 5**). The presence of specific bands in DGGE profiles of gut microbiota in litters belonging to sows treated with probiotic was evaluated using Pearson’s chi-squared test (χ^2^) where only the clearly visible bands were counted. Results of χ^2^ test revealed that proportions of six bands obtained with Eubacteria-specific primers were significantly higher (62.5–87.5%) (*p* < 0.05) and proportions of three bands obtained with the same primers were significantly lower (50–87.5%) (*p* < 0.05) in litters of probiotic-treated sows (**Table 1**). Additionally, the analysis detected two bands obtained with lactobacilli-specific primers that were present in significantly lower (50–62.5%) (*p* < 0.05) proportions in litters of probiotic-treated sows (**Table 1**).

Concerning bacterial diversity, results of *t*-test revealed significantly higher average number of bands obtained with Eubacteria-specific primers in litters of treated sows (39) as compared to litters of non-treated sows (32.75) (*p* < 0.05). No differences were obtained with LAB-specific primers. We additionally used matched statistical analysis to compare total bacterial diversity in litters with bacterial diversity in their mother sows, using Wilcoxon Signed Ranks test. This test revealed that probiotic treatment caused an increase of average number of Eubacteria-specific bands in litters (39) (*p* < 0.05) relative to their mothers before treatment (19.75). There were no differences between litters and their mothers in non-treated group. Furthermore, no differences were obtained with LAB-specific bands for none of the treatment groups (**Figure 5**). Hence, the results indicate that probiotic treatment of pregnant sows positively influenced the gut microbiota diversity of neonatal piglets.

## Discussion

In this study the potential beneficial effect of new fermented dairy probiotic culture based on three natural isolates *L. helveticus* BGRA43, *L. fermentum* BGHI14 and *S. thermophilus* BGVLJ1-44 was investigated. The main aim was to establish the gut health and promote gut colonization of neonatal piglets with beneficial bacteria by competitive colonization of probiotic strains before contact with pathogens from the environment.

A number of studies have been carried out in order to determine the efficiency of probiotics against intestinal infections in domestic animals (Kenny et al., 2011; Sornplang and Piyadeatsoontorn, 2016). Particularly, several microorganisms (bacteria and yeasts) were evaluated as potential probiotics for pigs (Tournut, 1989; Ezema, 2013). According to FAO/WHO (FAO-WHO, 2006), probiotics need to be non-pathogenic, non-toxic and have to exhibit beneficial effect on the host. Lactic acid bacteria are generally regarded as safe (GRAS). Moreover, in our previous studies the strains BGRA43 and BGHI14 were successfully used in animal experiments and determined as safe (Lukic et al., 2013; Strahinic et al., 2013), while the strain BGVLJ1-44 was isolated from artisanal yogurt and successfully used as starter culture for cheese manufacturing. In addition, all three strains are previously shown to be sensitive to the relevant antibiotics proposed by EFSA (European Food Safety Authority (EFSA), 2012; Golic et al., 2017).

In order to be effective in gut environment the probiotic should be capable to survive the GIT passage. Hence, the survival of the probiotic strains BGRA43, BGHI14 and BGVLJ1-44 in simulated GIT conditions was tested in this study. The obtained results suggested that all three strains could adequately survive the passage through stomach, while only two strains BGRA43 and BGVLJ1-44 survived the passage through dudenum (from initial 10^8^ CFU/ml to 10^7^ CFU/ml after incubation in gastric juice and bile salts; approximately 10% survival). Moreover, only the strains BGRA43 and BGVLJ1-44 successfully survived the conditions simulating colon environment (10^6^ CFU/ml; survival approximately 1–2%). The number of viable cells of strain BGHI14 was quite lower (10^4^ CFU/ml; survival approximately 0.01%), hence this strain possibly exhibit beneficial effect as postbiotic. The best survival ability exhibited the EPS-producing strain BGVLJ1-44, which could survive even when applied in saline buffer. It could be hypothesized that the EPS layer present on the surface of BGVLJ1-44 helps the strain to deal with the harsh conditions in the gut environment, similarly to the previously reported *Bifidobacterium animalis* subsp. *lactis* strains (de los Reyes-Gavilán et al., 2011). However, the survival of other two strains BGRA43 and BGHI14 was achieved only when bacteria were applied in 1% skimmed milk. It was previously reported that food carriers, such as milk, favors survival through the GIT due to the buffering and protective effect (Ranadheera et al., 2010; Nikolic et al., 2012).

The colonization of intestinal mucosa, persistence in the gut and competitive exclusion of pathogens are the important probiotic features, according to FAO/WHO criteria (FAO-WHO, 2006). In fact, the health-promoting effects of probiotics partly depend on their ability to adhere to gut mucosa and to neutralize the effects of pathogens (Collado et al., 2009). This property is strain-dependent and could be achieved by different mechanisms, e.g., a physical blocking of the pathogen’s binding to IEC (colonization competition), increasing the tight-junctions and reinforcing the permeability of the epithelium, induction of mucus production by IEC, as well as stimulation of the innate immune response (Gareau et al., 2010; Liévin-Le Moal and Servin, 2014). Moreover, the most promising property of probiotics used in piglets during the weaning period, is shown to be related to the ability to competitively exclude pathogenic (Angelakis, 2017). Thus, the ability of the strains and probiotic combination to competitively exclude *E. coli* ATCC25922 and *Salmonella* 654/7E was examined using Caco-2 cells. Here, we report that the strains used in this study, have moderate adhesion ability (7–15%) enabling the strains to colonize the intestinal mucosa. The results revealed that the strain BGRA43 would be the most capable to compete with pathogens, particularly *E. coli* and *Salmonella* sp., to colonize the intestinal environment. In addition, the strain BGHI14, exhibiting the lowest adhesion ability, significantly (*p* < 0.05) reduced the adhesion of *E. coli* ATCC25922. Hence, it could be concluded that this feature is not correlated with the adhesion ability similarly as shown for the strain *Lactobacillus paracasei* BGSJ2-8 (Živković et al., 2016). Interestingly, the EPS-producing strain BGVLJ1-44 most significantly (*p* < 0.01) diminished the association of *Salmonella* 654/7E to Caco-2 cells, possibly due to the presence of ropy EPS at the cell surface. The results are in accordance to the previous findings suggesting that *Salmonella* 654/7E cells could not access the intestinal mucosa due to physical blocking of bacteria in EPS matrix (Živković et al., 2016). Moreover, the results of Polak-Berecka et al. (2014) indicated that EPS produced by *L. rhamnosus* E/N hinder the adhesion of bacteria by masking the receptors at the host epithelial cell surface. However, the similar effect was not seen for *E. coli* ATCC25922 strain, pointing out that interactions between probiotics and pathogens are strain- (both probiotic and pathogen) dependent. The abilities of diverse probiotic strains to reduce the association of various pathogens to intestinal cells were previously reported, suggesting that this feature is most likely related to the presence of specific structural components on the bacterial cell surface including cell surface associated proteins, S-layer proteins, aggregation factors and EPS (Varma et al., 2010; Zhang et al., 2010; Kaewnopparat et al., 2013; García-Cayuela et al., 2014; Živković et al., 2015, 2016). Interestingly, the adhesion of *Salmonella* 654/7E in the presence of mixed probiotic culture was not reduced, indicating that possible antagonistic effects among the strains in probiotic mixture could be assumed.

Further, in this study we have treated the sows in the period of 10 days before the farrowing and the results revealed that the probiotic successfully reduced the number of viable cells of *Enterobacteriaceae* in litters of probiotic treated sows, confirming the results obtained in *in vitro* experiments showing ability of the probiotic strains to reduce association of *E. coli* and *Salmonella* to Caco-2 cells. Similarly, the literature data indicated that use of various lactobacillus probiotic strain *L. paracasei*, *L. sobrius*, *L. rhamnosus* GG decreased the count of *Enterobacteriaceae*, particularly *E. coli* and ETEC (Mozzi et al., 2015). In contrast, the treatment of grower and finisher pigs with various probiotic products based on *Bacillus* sp. showed inconsistent results, in one case *Bacillus* did not affect fecal LAB and *E. coli* counts, in other fecal LAB counts were increased while fecal coliform counts were not affected (Biomate 2B, Chr. Hansen Biosystems, Hoersholm, Danmark). When commercial product “Pelletmate livestock” (Chr. Hansen Biosystems) were supplemented, fecal coliform counts were decreased while LAB count was not affected, although increase in LAB, especially lactobacilli, was previously linked to health promoting effects in piglets and was inversely correlated with enterobacteria count (Pieper et al., 2009; Giang et al., 2011; Mozzi et al., 2015). Similarly to the results obtained for *Bacillus* sp. our results, obtained both by culture-dependent and DGGE method did not show significant differences in LAB counts between litters from treated and untreated sows. The discrepancy could be appointed to the fact that lactobacilli normally inhabit distal part of small intestine; hence the analysis of ileal microbiota would be needed to assess the influence of probiotic treatment of sows on total lactobacilli diversity in the litters (Tajima and Aminov, 2015).

Finally, the influence of probiotic on total microbiota diversity was evaluated and the significant increase of gut microbiota diversity in neonatal piglets was observed comparing to treated sows. The influence of probiotics on gut microbiota composition, diversity and function has been intensively studied recently, although the most data have been obtained in human studies (Hemarajata and Versalovic, 2013). For instance, DGGE analyses of human fecal microbiota of IBS patients revealed that microbiota composition was more similar in probiotics-treated patients than in the placebo group, suggesting that probiotics treatment stabilizes microbiota composition (Ki Cha et al., 2011). However, data related to influence of probiotics on piglets gut microbiota are limited. Recently, gnotobiotic piglets are used as a model to test the changes in gut microbiota composition in response to environmental factors. Similarly to the results of human studies, it was observed that the oral application of *L. rhamnosus* LGG in piglets prevented the changes in gut microbial composition caused by Human rhinovirus (HRV) infection (Zhang et al., 2014). The results of this study strongly indicate that gut microbiota diversity in piglets of treated sows is significantly increased comparing to their mother sows, while such increase was not scored in control groups.

## Conclusion

According to our knowledge, this is the first study reporting that application of probiotic on sows positively influences the gut microbiota diversity in piglets. The results obtained in this study indicate that treatment of sows with new fermented dairy probiotic provides successful colonization of the piglets’ gut with beneficial bacteria, improves the gut microbiota diversity and prevents the infection of neonatal piglets. Taking the results together it could be concluded that the probiotic would be a good candidate for application as feed additive in the pig industry for prevention of intestinal infections in neonatal piglets.

## Author Contributions

KV, AT-V, IM, and NG: conception and design of the study. KV, AT-V, and NG: performed the main work. SM and JB: participated in the research – microbiology. MD, JL, and MŽ: participated in the research – host microbe interaction. KV and MT: participated in the research – DGGE analysis. JL: participated in the research – statistical analysis. IM: participated in the research – farm trial. NG, KV, AT-V, SM, and JL: analyzed, interpreted, and critically revised the data. KV and NG, prepared the manuscript for submission. All authors finally approved the version to be published.

## Conflict of Interest Statement

The results presented in the manuscript are part of the national and PCT patent application submitted before the Intellectual Property Office, Serbia. The reviewer RT and handling Editor declared their shared affiliation.
